# The Greatest Show (Birds) on Earth: Public Presentations and Their Welfare Impacts on Zoo‐Housed Birds

**DOI:** 10.1002/zoo.70089

**Published:** 2026-07-01

**Authors:** Bridget Cooper‐Rogers, Paige Klingner, Sara Hanson, Justine Partoon, Eduardo J. Fernandez

**Affiliations:** ^1^ School of Animal and Veterinary Sciences University of Adelaide Adelaide South Australia Australia; ^2^ Adelaide Zoo Adelaide South Australia Australia

**Keywords:** animal interaction programs, animal welfare, animal‐visitor interactions, birds zoos

## Abstract

Animal interaction programs (AIPs) are a sub‐category of animal‐visitor interactions (AVIs) and are increasingly prevalent across accredited zoos worldwide. Although AVIs are being increasingly researched, few studies have explored the welfare implications of AIPs, such as the potential animal welfare impacts of public feedings or public presentations. Bird shows and presentations are among the most2021a advertised AIPs within zoological institutions yet suffer from a lack of empirical studies. This study investigated behavioral and physical measures to assess the welfare of several bird species located at Adelaide Zoo. An ethogram was developed to record behaviors across three comparisons: (1) show days versus non‐show days, (2) before and after shows, and (3) show birds versus non‐show birds. In addition, feather and body condition scores were recorded and compared across show birds and non‐show birds. Multiple comparisons were provided in an attempt to avoid limited and context‐specific data, which assisted in a more comprehensive and well‐rounded welfare assessment. Show birds demonstrated an increase in abnormal behaviors on show days compared to non‐show days, which prompted further comparisons to determine why the increased prevalence of abnormal behaviors was occurring on the days a presentation took place. On presentation days, show birds demonstrated more active, social, and abnormal behaviors pre‐show but exhibited an increase in grooming, foraging, and a significant reduction in abnormal behaviors post‐show, which could suggest a potential anticipatory effect. In addition, show birds had a closer‐to‐ideal body condition score and demonstrated significantly less inactive and abnormal behavior compared to non‐show birds. The results suggested that public bird shows and presentations have a largely minimal or neutral welfare impact, however, there may be a potential enriching aspect of bird shows and presentations.

## Summary

- Minimal literature has explored the welfare impacts of the animals involved in animal interaction programs within zoos.
- Behavioral and physical measures were used to assess show bird welfare and make comparisons against show days and non‐show days, pre and post‐ show, and show birds to non‐show birds.
- Results indicated that shows had a largely neutral welfare impact and a potentially enriching effect on the birds involved.

## Graphical abstract

Birds involved in bird shows were compared against non‐show birds regarding show paticipation, behaviour, body score condiiton, and feather condition. Additionally, comparisons between on and off show days as well as before and after the show were made to investigate differences in behaviour and condition. Show birds demonstrated a closer to ideal body condition score as well as reduced inactivity and abnormal behaviours. Anticipatory behaviours were present. Public shows had no overall negative welfare impact on the birds in the program. However, may have a potentially enriching effect that needs to be studied in more detail.

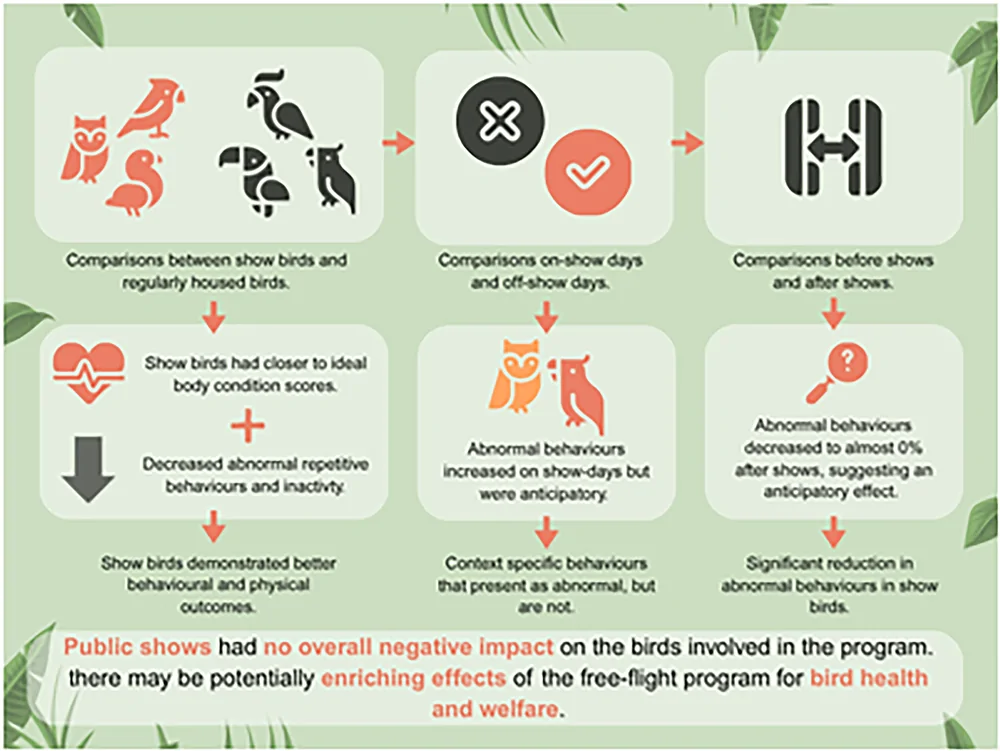

## 1 Introduction

Animal interaction programs (AIPs) are a common practice across zoos worldwide, with over 75% of accredited zoos featuring at least one (D'Cruze et al. 2019; Spooner, Jensen, et al. 2021). AIPs are a sub‐category of animal‐visitor interactions (AVIs), with AVIs gaining research traction over the past few decades within zoos and aquariums (Learmonth et al. 2021; Sherwen and Hemsworth 2019; Spooner, Farnworth, et al. 2021). AIPs can be defined as a planned interaction between a visitor and an animal, typically for education or conservation purposes (Fernandez et al. 2009; Kreger and Mench 1995; Spooner, Farnworth, et al. 2021). These interactions can be categorized as direct or indirect; direct interactions include any physical contact with an animal, such as some animal ambassador programs or public feedings, while indirect interactions refer to non‐contact experiences such as visitors attending or observing an animal demonstration (Clifford‐Clarke et al. 2022; D'Cruze et al. 2019; Whitehouse‐Tedd et al. 2021).

AIPs are crucial to most accredited zoo goals, as they have been shown to incite conservation action in visitors through recurring visits, donations, and purchasing merchandise (Collins et al. 2023; Godinez and Fernandez 2019; McNally et al. 2024; Patrick et al. 2007). Furthermore, AIPs have been documented to improve visitor knowledge and understanding of related conservation topics and promote positive experiences with the animals involved. Visitor participation in these programs has demonstrated a higher efficacy on retained knowledge regarding conservation education post‐experience (Clayton et al. 2008; Spooner, Jensen, et al. 2021). These programs have been shown to engage visitors for longer periods compared to other informational graphics and allow visitors to create stronger emotional connections toward the animals involved in these programs and facilitate positive attitudes toward conservation action (D'Cruze et al. 2019; Lyon et al. 2023; Patrick et al. 2007). Education and conservation are key goals that accredited zoos strive to promote, which highlights the importance of involving visitors in AIPs, as they not only enhance but also cultivate the visitor's ability to learn (Collins et al. 2023; Learmonth et al. 2021; Spooner, Jensen, et al. 2021).

Although it is documented that visitors benefit from participating in AIPs, little is known about the impact of AIPs on the animals involved in these programs. From prior literature, three distinct visitor effects on welfare have been categorized: (1) positive, (2) neutral, or (3) negative (Fernandez and Chiew 2021; Hosey 2000; Hosey 2008). However, these results have been incredibly varied depending on the species, location, the type of program, and even at an individual level (Fernandez and Sherwen 2024; Hosey 2008; Powell et al. 2020).

Ensuring high welfare standards and promoting positive welfare is a priority for zoos, with research playing a key role in this achievement (Hill and Broom 2009; Maple and Perdue 2013). However, there is an inherent bias toward mammalian species in zoo welfare literature. For instance, from 2008 to 2017 mammalian welfare literature consisted of 75% of all zoo welfare research, while bird welfare literature made up only 10% (Binding et al. 2020). Furthermore, birds have been documented to make up more than 58% of species in zoos compared to 25% of mammals, which demonstrates a disparity and underrepresentation of bird welfare within current zoo welfare literature (Conde et al. 2013; Binding et al. 2020; Woods et al. 2022). Although literature is consistently increasing the variety and species of taxa studied, there is an indisputable gap regarding bird welfare research in zoos (Binding et al. 2020; Woods et al. 2022). In addition, birds are the most common taxa involved in shows or demonstrations, with 52% of all animal presentations consisting of bird species (D'Cruze et al. 2019). However, less than 2% of zoo welfare literature has investigated the welfare impacts of AIPs, specifically shows, presentations, and ambassador programs, which highlights a large gap in understanding not only how AIPs impact animal welfare but particularly in taxa other than mammals (Binding et al. 2020; Woods et al. 2022).

There is a substantial lack of understanding regarding the welfare impacts that AIPs can potentially have on the animals participating. Current literature has emphasized various and mixed welfare outcomes due to involvement in AIPs, which could potentially differ due to factors such as the type of AIP, the species involved, and other individual factors (Hartell‐DeNardo et al. 2023; Kozlowski et al. 2021; Webster et al. 2017). A review regarding ambassador animal programs by Spooner, Jensen, et al. (2021) emphasized that the current zoo welfare literature that has investigated the potential welfare impacts of being involved in animal ambassador programs is too inconclusive to denote a singular welfare impact. Although ambassador programs are a type of AIP and do not represent all AIPs, this trend has been seen amongst other AIP literature, in which welfare outcomes are undetermined or mixed (i.e., a neutral and positive outcome). For instance, Kyngdon et al. (2003) observed that dolphins (*Delphinus delphis*) involved in swimming with visitor experiences utilized refuges more when visitors were present, with some dolphins displaying increased abrupt and aggressive behaviors after sessions and others displaying increased touch and reduced aggression during sessions, while Trone et al. (2005) observed an increase in play behaviors in bottlenose dolphins (*Tursiops truncatus*) after swimming with visitors. Furthermore, H. Jones et al. (2016) observed that crowned lemurs (*Eulemur coronatus)* that participated in public feedings interacted more with visitors and displayed less aggressive behaviors, while giraffes involved in a public feeding program had no change in the expression of abnormal behaviors, however, feeding increased, which may have caused a reduction in the expression of oral abnormal behaviors (Orban et al. 2016). Regarding an article that investigated the handling of ambassador animals, Baird et al. (2016) observed several species of armadillos, hedgehogs, as well as red‐tailed hawks and determined that handling did not impose negative welfare impacts. However, the duration of handling was associated with an increase in Fecal Glucocorticoid Metabolite levels, which could be an indicator of stress. Fernandez et al. (2021a) observed that both African and Asian elephants (*Loxodonta Africana* and *Elephas maximus indicus)* involved in public feeding demonstrations displayed increased species‐specific behaviors, including social, active, and foraging behaviors, as well as a reduction in abnormal behaviors post‐session. These examples highlight the complexity of assessing and interpreting AIP welfare literature, as there is no single answer or comparisons to how welfare states are impacted across the type of AIP, animal involvement, species, differing ecologies, and even across individuals.

To the author's knowledge, there are no current papers that look specifically at the welfare impact of public shows or presentations within bird species in zoos. The current literature that has investigated avian species involved in AIPs has explored ambassador, feeding, and walk‐through aviary programs (Baird et al. 2016; Blanchett et al. 2020; Hartell‐DeNardo et al. 2023; Ramont et al. 2021; Saiyed et al. 2019). These programs emphasized various welfare impacts, with most having neutral or minimal outcomes, although positive and negative outcomes were also observed. It is hypothesized that, similarly to these studies, minimal welfare impacts will be observed in birds participating in public performances or shows.

The following study examined the impacts of bird presentations on the welfare of the birds involved in those shows or presentation programs. There were three aims in relation to this study: (1) establish the impact that presentations have on multiple welfare‐related metrics in show birds; (2) investigate welfare differences between show birds and regularly housed birds; and (3) better understand measures associated with positive and negative welfare in zoo‐housed birds.

## 2 Materials and Methods

### 2.1 Subjects

Prior to commencing observations, ethics approval was granted by the University of Adelaide Animal Ethics Committee (S‐20240037), and all methods were in accordance with the Australian code for the care and use of animals for scientific purposes, 8th edition (National Health and Medical Research Council: Canberra, 2013). A total of 7 bird species and 10 individuals were involved, as illustrated in Table 1. All the birds were DNA sexed by Adelaide Zoo staff prior to the study, and each of the birds ranged from 3 to 20 years old. However, only estimated ages could be provided as most of the individuals were rehabilitated from the wild.

**Table 1:** The birds involved in the study are listed, including species, number of individuals, show status, and sex. If a bird participated in the free‐flight show then that was denoted as “involved,” if a bird did not participate in or was associated with the show at all, that was noted as “not involved.”

Name	Species	Show involvement	Sex
Owlbert	Barking owl (*Ninox connivens*)	Involved	Female
Oscar	Sooty owl (*Tyto tenebricosa)*	Involved	Male
Buzz	Black‐breasted buzzard (*Hamirostra melanosternon*)	Involved	Female
Digit	Nankeen kestrel (*Falco cenchroides*)	Involved	Female
Manu	Blue and gold macaw (*Ara ararauna*)	Involved	Male
Kath	Galah (*Eolophus roseicapilla*)	Involved	Female
Spence	Red‐tailed black cockatoo (*Calyptorhynchus banksia*)	Involved	Female
Kim	Galah (*Eolophus roseicapilla*)	Not involved	Female
Storm	Red‐tailed black cockatoo (*Calyptorhynchus banksia*)	Not involved	Female
Banks	Red‐tailed black cockatoo (*Calyptorhynchus banksia*)	Not involved	Male

### 2.2 Setting

All species were housed at Adelaide Zoo, Zoos South Australia, and were kept in a row of large aviaries behind the free‐flight stage that ranged from ~3.9 to 20.8 m × 2.3 to 10.4 m × 2.6 to 2.8 m. Most of the birds were rotated through aviaries daily, excluding the sooty and barking owl. Conspecifics were housed together, regardless of show involvement, and no cross‐species housing occurred. Three of the seven species (black‐breasted buzzard, barking owl, and Nankeen kestrel) were viewable by the public, while the other species were housed in a staff‐only area. The staff‐only area consisted of a corridor with several holding aviaries and was undercover with access to natural light. The corridor allowed easy access for transportation to and from the presentation stage as well as routine husbandry procedures such as food preparation, cleaning, enrichment, and training.

All species received enrichment daily via an enrichment rotation schedule that included cardboard boxes, commercial bird toys, swings, and puzzle feeders. In addition, parrot species were also provided with branches of native Australian trees daily. The three aviaries that faced the public were naturalistic and planted with varied species of trees, shrubs, and bushes, as well as decorated with rocks and foliage. Whereas the holding aviaries in the staff‐only area had smooth concrete flooring for ease of daily cleaning but featured native tree branches and plants to mimic a natural habitat.

Prior to the presentation, show birds were fed and weighed by staff from approximately 08:30–09:00 h. The parrot species received Wombaroo parrot pellets, seasonal fruits and vegetables, and various nuts and seeds. Whereas the raptor species were fed a variety of mice, quails, and day‐old chicks. During the free‐flight program, the parrots received nuts and seeds as a food reward, while the raptor species were fed small portions of mice.

The back of the off‐exhibit aviaries faced the show stage, which consisted of a platform and a wooden stage with several natural wooden perches adjacent to a lawn oval. The presentation was announced over the public address systems at 11:15 h daily in preparation for the show, which began at 11:30 h. To optimize welfare practices, the birds were provided with a choice of whether to participate in the show or not by their responses to staff. If a bird did not fly over when prompted, that was recognized as choosing to not participate, while flying over to a staff member was recognized as choosing to participate.

The free‐flight show consisted of a staff‐led educational talk about each of the species, their natural habitats, and other “fun facts” to the public while demonstrating various natural behaviors such as flying from point A to point B (all species) or cracking open a plastic emu egg with a rock for a food reward (black‐breasted buzzard). The staff member would attend to the birds' responses throughout the presentation, and as such, if any signs of stress or discomfort were displayed (e.g., head tossing), the presentation would be adjusted or concluded early to suit the needs of the birds.

### 2.3 Materials

Eight cameras from Swann Security Systems were numbered and positioned in front of each of the seven aviaries. Each of the cameras was secured with zip‐ties and angled to ensure that most of the aviary was visible. The eighth camera was positioned to view the top corner of one of the raptor's enclosures, as it was much larger than the other aviaries, and staff had identified that there was a preferred perching area there. The cameras were attached by cables that were plugged into a channel box and a Dell monitor, which displayed live camera feeds. The cameras were set to record an hour before (10:00–11:00 h) and after (12:00–13:00 h) shows, which ran from 11:15 to 12:00 h daily. Approximately 30 min prior to the bird show, the show birds were taken out of the aviaries and out of view of the cameras and placed in holding aviaries in preparation for the show. The recordings were exported to a 2TB external hard drive weekly, and handheld tablets with ZooMonitor (Wark et al. 2019) were used to collect and code all behavioral data via video.

### 2.4 Data Collection and Procedure

A Body Condition Score (BCS) chart (see Figure 1) was designed based on previous work by Clements and Sanchez (2015) and Meredith (2022). The BCS chart was designed to visually assess fat and muscle distribution. As per standardized BCS charts, a 1–5 numerical scale was utilized, in which 3 was “ideal,” and 1 and 5 were denoted as extreme. A Feather Condition Score (FCS) chart (see Table 2) based on prior work by Meehan et al. (2003) and Kalmar (2011) was devised to visually assess plumage health and was rated on a numerical scale from 1 to 5, with 1 being ideal and 5 being extreme.

**Figure 1:**
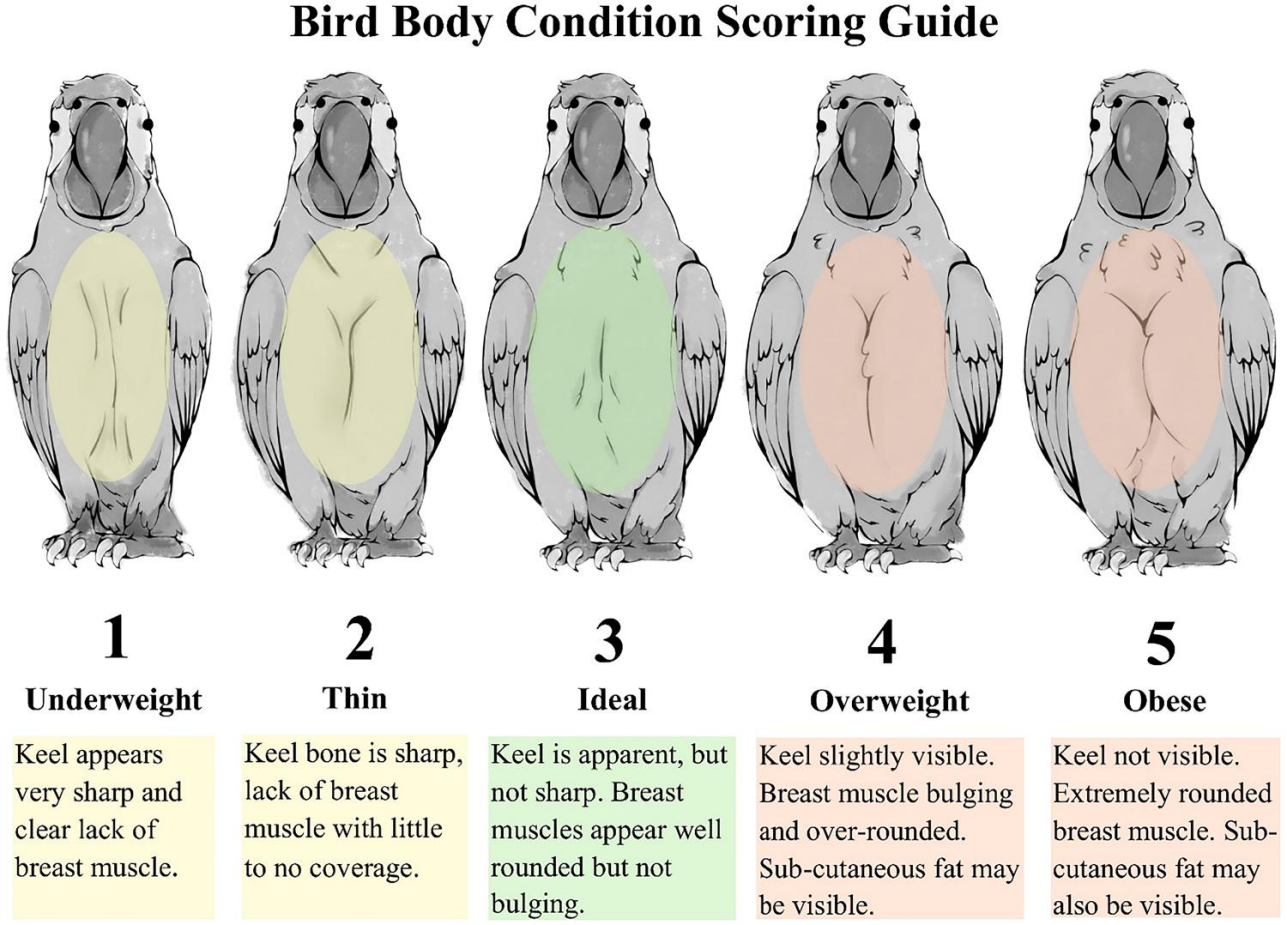
Body Score Condition chart to visually assess the body condition of the birds included in the study. Adapted from preliminary observations, Clements and Sanchez (2015), and Meredith (2022).

**Table 2:** Feather Condition Score chart to visually assess the plumage of the birds in the study. Adapted from preliminary observations, Meehan et al. (2003), and Kalmar (2011).

Feather condition score	Description
1	All feathers intact, no dirt or soilage. Glossy and bright feathers. Little to no fraying of feathers. No down feathers visible or skin damage.
2	Some feathers removed, soiled, frayed, or broken. Minimal exposure of down feathers.
3	Less than half of feathers removed. Some feathers dirty, none soiled. Some down feathers exposed, no evidence of skin or tissue injury.
4	More than half of feathers removed. Most feathers dirty or partly soiled. Most down feathers exposed, some evidence of skin or tissue injury.
5	All feathers removed. All feathers dirty or soiled. Skin exposed, clear evidence of skin or tissue injury.

An ethogram (Table 3) was devised based on preliminary observations and current peer‐reviewed literature by Smith and Wassmer (2016) and Rose et al. (2018), The ethogram was designed to be mutually exclusive, exhaustive, and welfare‐related, with a focus on commonly observed behaviors, and was split into seven behavioral categories (Active, Foraging, Social, Grooming, Inactive, Abnormal, and Other). It is additionally worth noting that more than 99% of instances from the “Other” category were out of sight.

**Table 3:** A total of 19 behaviors and seven behavioral categories were used to document behaviors on ZooMonitor. Adapted from preliminary observations, Rose and colleagues (2018), and Smith and Wassmer (2016).

Behavioral class and behaviors (abbreviated)	Definition
Active	
Locomotion (Lo)	Directed non‐repetitive movement utilizing legs to move from point A to point B, excluding flight and climbing.
Flying (Fl)	Wings are used (flapping/gliding) while entire body is airborne.
Climbing (Cl)	Claws and beak used to move on an exhibit structure (takes priority over flapping or locomotion).
Flapping	Fully extended, repeated wing movement without body movement or airborne body.
Foraging	
Eating/drinking (ED)	Beak contact with anything edible (not excrement or enrichment), including water.
Pecking (Pe)	Using beak to contact non‐edible, non‐body items.
Manipulating object (MO)	Contact with a non‐edible object (e.g., enrichment), with any part of the body.
Social	
Interacting w/other animal (IOA)	Any physical contact with another bird, inclusive of allopreening and aggression.
Interacting w/staff (IP)	Any physical contact with a staff member.
Groom	
Preening/scratching body (PSB)	Using beak or claws to rub/contact own feathers, as well as shaking/fluffing feathers (end of preening).
Bathing (Ba)	Submerging part of body in water (without drinking) or dirt and repeatedly shaking, while raising feathers and wings (often covered in dust or dipping in water).
Inactive	
Perching/standing (St)	No movement, body with upright position (at least one foot clearly visible).
Resting (Re)	No movement with legs tucked under body (not visible), often with one or both eyes closed.
Abnormal	
Repetitive behavior (RB)	Repeated pattern of behavior that involves at least one back‐forth‐back motion (e.g., pacing from point A‐B‐A, feather plucking, self‐mutilation).
Coprophagy (Co)	Beak contact with excrement.
Other	
Defecating (De)	Excreting waste.
Out of Sight (OS)	Not visible to observer.
Other (Ot)	Engaged in an activity that does not meet one of the behaviors listed above.

Individuals were housed with conspecifics for welfare reasons as determined by Adelaide Zoo prior to the study. Staff used physical characteristics to distinguish between individuals, which were relayed to observers and included banding, sexual dimorphism, and key physical characteristics (e.g., missing a wing). Subjects were organized into two categories, (1) show or (2) non‐show, based on their involvement in the public presentation (see Table 1) prior to the study by the zoo staff. The ‘show’ group consisted of birds that were involved in and participated in the free‐flight show, as determined by Adelaide Zoo staff prior to the study, whereas the “non‐show” group was composed of birds that were not involved and did not participate in the free‐flight presentations. The study was quasi‐experimental in that the staff determined how the groups were selected and therefore had a semi‐randomized design set of reversals, which focused on three comparisons: (1) show days versus non‐show days, (2) before versus after shows, and (3) show birds versus non‐show birds. The first and second comparisons were within‐subject, while the tertiary comparison was between‐subjects. All birds were observed for 1‐h sessions twice per day, 6 days a week. Sessions ranged from 50 to 112 per show bird, with variation occurring due to daily aviary rotation, husbandry, and veterinary requirements. The Nankeen kestrel was removed after 50 sessions from both the free‐flight program and aviary due to veterinary requirements and was not included in further observations. For the show birds, a total of 713 sessions were collected, with 507 show sessions and 206 non‐show sessions. In addition, a total of 336 sessions were collected for the non‐show birds.

The study spanned over a period of 64 days (July 1, 2024 to September 3, 2024). A participation form for the birds involved in the show (Appendix 1) was created for staff to note whether a show bird did or did not participate in the presentation that day, alongside correlating dates. Prior to behavioral observations, show birds were categorized into “yes” or “no” depending on presentation participation for that day, and BCS as well as FCS were scored and recorded in Microsoft Excel daily. Observations took place utilizing ZooMonitor and consisted of 1‐h sessions with 5‐min intervals. Instantaneous time (pinpoint) scan sampling was used across birds, hence why both state and event behaviors are included within the ethogram, and all birds were recorded (scanned) for their behavior at each interval (Brereton et al. 2022; Bateson and Martin 2021).

### 2.5 Interobserver Agreement

For 32.1% of the data collection period, a second observer was present to code data with the primary observer to assess reliability. Inter‐observer agreement was calculated based on the total agreement of three measures: each behavioral category (ethogram), BCS, and FCS. Total agreement was calculated using the following equation (Cooper et al. 2019):

$$\frac{\mathrm{Smaller}\;\mathrm{count}}{\mathrm{Larger}\;\mathrm{count}}\times \;100=\mathrm{total}\;\mathrm{count}\;\mathrm{IOA}\%$$

The average agreement across all behavioral categories was 85.9%, with each behavioral category scored as follows: Active (89.4%), Foraging (89.3%), Social (83.6%), Grooming (77.7%), Inactive (90.3%), Abnormal (82%), and Other (89%). Body condition as well as FCS had an average agreement of 94.9% and 95%, respectively. The disparities largely arose from the initial sessions as the second observer was learning to identify conspecifics (e.g., the two female galahs). This was mitigated in future sessions by engaging in further training on identifying key characteristics.

### 2.6 Statistics

The raw data were downloaded from ZooMonitor to collate and organize in Microsoft Excel, which was used to produce all descriptive statistics, including the mean and standard error of the mean (SEM). GraphPad Prism version 10.1.0 was utilized for all statistical analyses and for producing graphs. Shapiro–Wilk tests determined that all the data failed normality; consequently, Mann–Whitney *U* tests were run to analyze the data. Bonferroni corrections were used to reduce false positives by dividing α = 0.05 by the number of behavioral categories (0.05/7), which set the *p* value significance level at 0.007. Behavioral categories instead of individual behaviors were analyzed for significant differences in each of the three comparisons.

## 3 Results

### 3.1 Show Birds: Show Days vs. Non‐Show Days

Figure 2 demonstrates the differences in show days (*n*_1_) versus non‐show days (*n*_2_) across the seven behavioral categories. A show day was denoted as a day in which a show bird participated in the free‐flight show, whereas a non‐show day was when a show bird did not participate in the show; hence, there is no data for non‐show birds within this section. Significant and nonsignificant differences are detailed, as well as the mean and SEM for each category and condition. Inactive behaviors were significantly lower on show days compared to non‐show days (*U* = 40561, *p *≤ 0.0001, *n*_1_ = 507, *n*_2_ = 206; show days: 33.09% ± 1.41; non‐show days: 47.98% ± 2.59). Abnormal behaviors were significantly higher on show days compared to non‐show days (*U* = 46648, *p* = 0.0011, *n*_1_ = 507, *n*_2_ = 206; show days: 4.64% ± 0.54; non‐show days: 2.92% ± 0.50). Other behaviors were significantly higher on show days compared to non‐show days (*U* = 44196, *p* = 0.0009, *n*_1_ = 507, *n*_2_ = 206; show days: 30% ± 1.38; non‐show days). Social behaviors approached significance but were not significantly different with the correction (*U* = 48,294, *p* = 0.0324, *n*_1_ = 507, n_2_ = 206; show days: 2.94% ± 0.26; non‐show days: 2.35% ± 0.43). There were no significant differences in Active behaviors (*U* = 47,890, *p* = 0.0761, *n*_1_ = 507, *n*_2_ = 206; show days: 18.34% ± 0.80; non‐show days: 17.15% ± 1.41). There were also no significant differences observed in Foraging behaviors (*U* = 51,443, *p* = 0.7064, *n*_1_ = 507, *n*_2_ = 206; show days: 7.20% ± 0.65; non‐show days 6.35% ± 0.92), or Grooming behaviors (*U* = 49,982, *p* = 0.1792, *n*_1_ = 507, *n*_2_ = 206; show days: 3.12% ± 0.34; non‐show days 2.18% ± 0.42).

**Figure 2:**
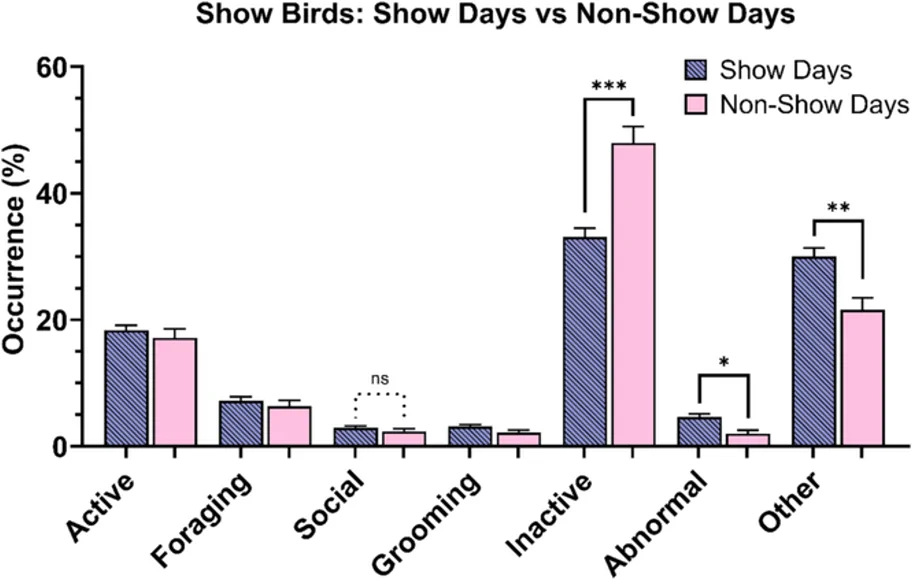
Show birds on show days vs. non‐show days. The graph demonstrates the mean percent (with SEM bars), with the stars and lines representing significant differences (* ≤ 0.001, ** ≤0.001, *** ≤ 0.0001). The dotted lines and “ns” represent data that were below 0.05 but not below the α = 0.007 set by the Bonferroni correction.

### 3.2 Show Birds: Before vs. After Shows

Figure 3 demonstrates the differences in times before a show (*n*_1_) versus after a show (*n*_2_) across the seven behavioral categories. Before a show was denoted as an hour prior to the show occurring, whereas after a show was noted as an hour after the show, this analysis only included days in which a show bird participated in a show, and thus only included show bird data. Significant and nonsignificant differences are detailed, as well as the mean and SEM for each category and condition. Social behaviors were also significantly higher before a show compared to after a show (*U* = 27,765, *p* < 0.0005, *n*_1_ = 252, *n*_2_ = 255; before show: 3.44% ± 0.34; after show: 2.45% ± 0.38). Grooming behaviors were significantly lower before a show compared to after a show (*U* = 28,390, *p* < 0.0001, *n*_1_ = 252, *n*_2_ = 255; before show: 0.69% ± 0.19; after show: 5.52% ± 0.62), as were Inactive behaviors (*U* = 21,443, *p* < 0.0001, *n*_1_ = 252, *n*_2_ = 255; before show: 22.95% ± 1.56; after show: 43.10% ± 2.16). Abnormal behaviors were significantly higher before a show compared to after a show (*U* = 21,741, *p* < 0.0001, *n*_1_ = 252, *n*_2_ = 255; before show: 8.70% ± 1.01; after show: 0.62% ± 0.15), as were Other behaviors (*U* = 18,517, *p* < 0.0001, *n*_1_ = 252, *n*_2_ = 255); before show 39.55% ± 1.80; after show: 20.56% ± 1.93), and Foraging behaviors (*U* = 28,234, *p* < 0.0056, *n*_1_ = 252, *n*_2_ = 255; before show: 4.46% ± 0.57; after show: 9.90% ± 1.14). There were no significant differences in Active behaviors (*U* = 28,113, *p* = 0.0132, *n*_1_ = 252, *n*_2_ = 255; before show: 19.41% ± 1.06; after show: 17.29% ± 1.20).

**Figure 3:**
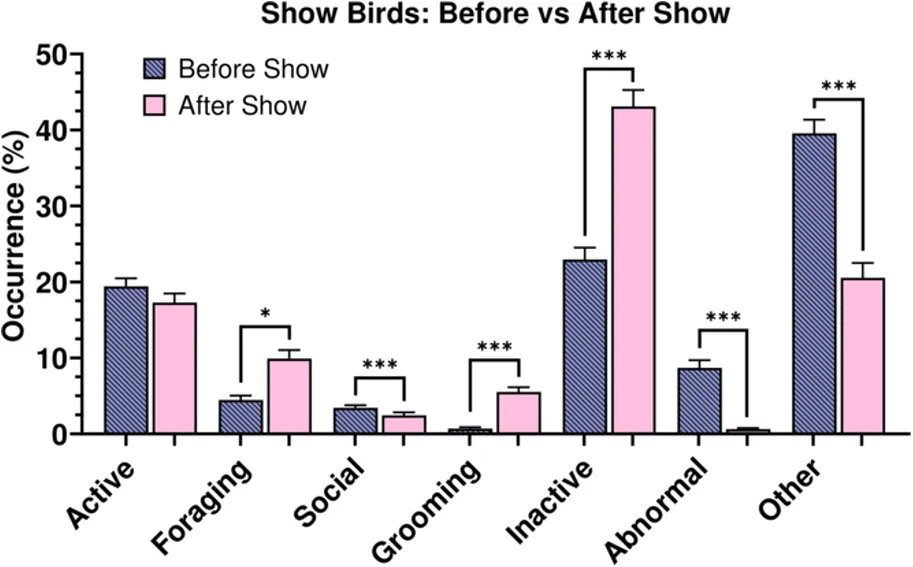
Before a show against after a show comparisons, on days in which a show did occur. The graph demonstrates the mean percent (with SEM bars), with the stars and lines representing significant differences (* ≤ 0.007, *** ≤ 0.0001). The dotted lines and “ns” represent data that were below 0.05 but not below the α = 0.007 set by the Bonferroni correction.

### 3.3 Show Birds vs. Non‐Show Birds: Behavioral Measures

Figure 4 demonstrates the differences in total show bird sessions in which one session equals one show day (*n*_1_) versus non‐show bird sessions (*n*_2_) across the seven behavioral categories. A show bird was a bird that was involved in and participated in the free‐flight bird show, whereas a non‐show bird was housed in the same aviary as the show birds but was not involved in and did not participate in the show. Significant and nonsignificant differences are detailed, as well as the mean and SEM of the mean for each category and condition. Foraging behaviors occurred significantly less in show birds compared to non‐show birds (*U* = 104,803, *p* = 0.0001, *n*_1_ = 713, *n*_2_ = 336; show birds: 6.95% ± 0.53; non‐show birds: 10.10% ± 0.87), as did Social behaviors (*U* = 108,608, *p* = 0.0014, *n*_1_ = 713, *n*_2_ = 336; show birds: 2.77% ± 0.22; non‐show birds: 4.22% ± 0.40), Inactive behaviors (*U* = 106,873, *p* = 0.0046, *n*_1_ = 713, *n*_2_ = 336; show birds: 37.39% ± 1.28; non‐show birds: 41.29% ± 1.54), and Abnormal behaviors (*U* = 109,619, *p* = 0.0021, *n*_1_ = 713, *n*_2_ = 336; show birds: 3.88% ± 0.41; non‐show birds: 4.22% ± 0.40). Other behaviors occurred significantly more in show birds compared to non‐show birds (*U* = 104,040, *p* = 0.0003, *n*_1_ = 713, *n*_2_ = 336; show birds: 27.56 ± 1.14; non‐show birds: 22.12% ± 1.63). Active behaviors approached significance; however, they were not significant with the correction (*U* = 110,565, *p* = 0.0397, *n*_1_ = 713, *n*_2_ = 336; show birds: 18% ± 0.70; non‐show birds: 14.06% ± 0.74). Grooming behaviors were not significant (*U* = 11,4650, *p* = 0.1048, *n*_1_ = 713, *n*_2_ = 336; show birds: 2.85% ± 0.27; non‐show birds: 3.00% ± 0.36).

**Figure 4:**
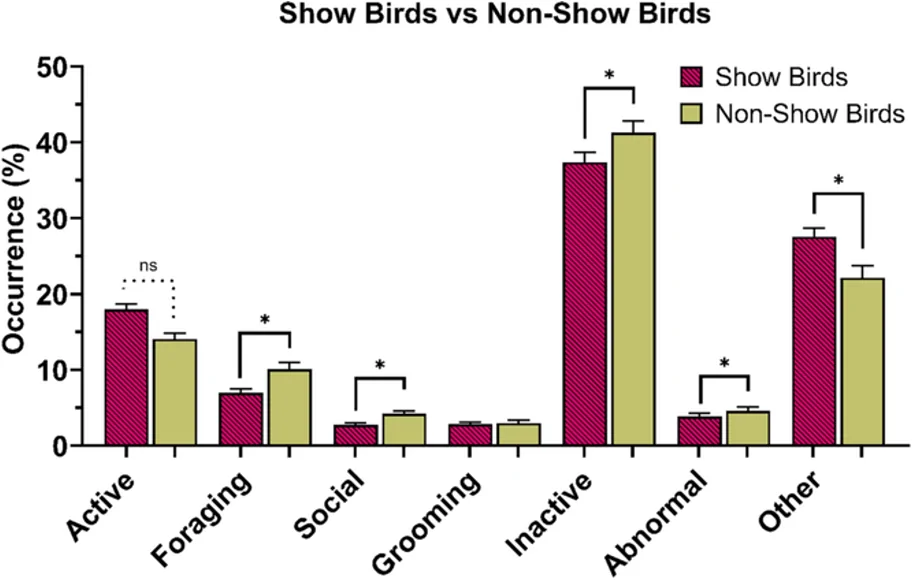
Show birds compared to the regularly housed “non‐show” birds. The graph demonstrates the mean percent (with SEM bars), with the stars and lines representing significant differences (* ≤ 0.007). The dotted lines and “ns” represent data that were below 0.05 but not below the α = 0.007 set by the Bonferroni correction.

### 3.4 Show Birds vs. Non‐Show Birds: Physical Measures

Figure 5 demonstrates the differences in BCS (left side of the figure) and FCS (right side of the figure) for show birds (*n*_1_) and non‐show birds (*n*_2_). There was a significant difference in BCS, with show birds being closer to the set ideal score of “3” (*U* = 29,866, *p* = 0.0039, *n*_1_ = 392, *n*_2_ = 171; show birds: 2.86 ± 0.02; non‐show birds: 2.75 ± 0.04). However, although show birds were closer to the set ideal of “1,” there were no significant differences in FCS (*U* = 31,936, *p* = 0.1348, *n*_1_ = 392, *n*_2_ = 171; show birds: 1.12 ± 0.02; non‐show birds: 1.17 ± 0.03).

**Figure 5:**
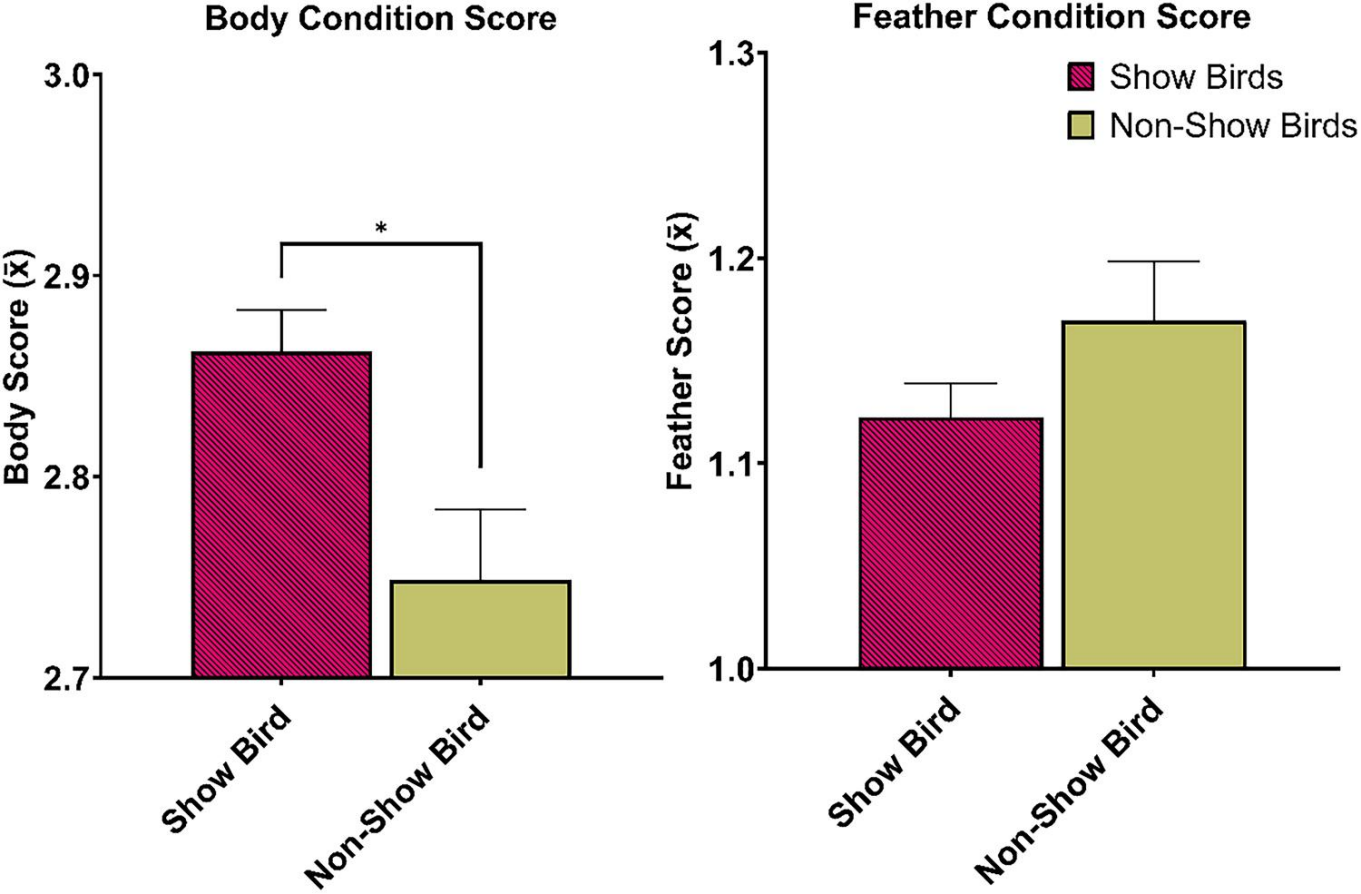
The body condition and feather condition score of show against non‐show birds. The graph demonstrates the mean percent (with SEM bars), with the stars and lines representing significant differences (* ≤ 0.007). The dotted lines and “ns” represent data that were below 0.05 but not below the α = 0.007 set by the Bonferroni correction. The ideal body score condition is 3, while the ideal feather condition is 1.

## 4 Discussion

### 4.1 Show Birds: Show Days vs. Non‐Show Days

All birds demonstrated a varied behavioral repertoire, with each behavioral class occurring at least once. Show birds demonstrated significantly less inactive behavior on presentation days, which could be indicative of a potential positive indicator of welfare. Wild parrot species have been documented to spend 45%–75% of their time exhibiting active behaviors such as foraging and traveling, which implies that inactivity is not a prevalent behavior within parrots (Brereton and Rose 2019; Magrath and Lill 1983; Rozek et al. 2010; Westcott and Cockburn 1988). The reduction in inactivity could imply that birds were engaging in alternative behaviors, such as species‐typical behaviors, which may have been attributed to the increase in social behaviors. Although this could suggest potential welfare benefits, it is hard to infer as the increase in social behaviors only approached significance and no other species‐typical behaviors were observed to increase (Brereton and Rose 2019; Clubb and Mason 2007; Fureix and Meagher 2015). A higher prevalence of inactive behaviors has been associated with lower arousal levels and poorer welfare in captive settings, which could indicate that a reduction in inactive behaviors is a positive welfare indicator in parrot species (Fureix and Meagher 2015; Less et al. 2012; Manteca et al. 2016; Tallo‐Para et al. 2023). However, inactivity alone cannot be used to assess welfare as context must be considered (Sherwen and Hemsworth 2019; Veasey et al. 1996). Depending on the species, topography, and species‐specific behaviors, inactivity can be interpreted to have multiple welfare outcomes and should be interpreted with caution. Although a reduction in inactivity may be beneficial for parrot species, it may have minimal welfare impacts on raptor species. In addition, in comparison to parrot species, raptors have differing ecologies, often spending higher proportions of time inactive (perching) in the wild during non‐breeding seasons and a higher proportion of time hunting or foraging throughout breeding seasons (Baladrón et al. 2016; Baladrón et al. 2018; Rijnsdorp et al. 1981). In addition, the “Other” category (out of sight) was the second most prevalent behavior on presentation days and may explain the reduction in inactivity, as the birds were not visible to the observer. A potential explanation for the increase in what was predominantly “out of sight” behavior was due to birds being removed from the aviaries at various points for routine husbandry, training procedures, and presentation preparation prior to the show.

There was a significant increase in abnormal behaviors on presentation days compared to days in which a presentation did not occur. Abnormal behaviors are typically utilized as markers of negative welfare and often arise from a lack of choice and control within the environment (Bacon 2018; Manteca et al. 2016; Tallo‐Parra et al. 2023; Wolfensohn et al. 2018). The increase in abnormal behaviors could potentially indicate that show birds were experiencing increased stress on presentation days. However, an increase in abnormal behaviors can also be related to anticipatory staff‐ or food‐seeking behaviors as other literature has described (Clegg et al. 2018; Krebs et al. 2017). Anticipatory behaviors have been defined as a goal‐oriented increase in behavior in response to a predictable event, such as routine feedings, interactions, or public presentations (Spruijt et al. 2001; van der Harst et al. 2003). As the free‐flight show occurred at the same time each day, this fits the definition of a predictable event, potentially introducing an anticipatory effect on the birds involved, which was observed as an increase in otherwise abnormal behaviors (Krebs et al. 2022; Jensen et al. 2013; Watters 2014). To establish whether the behaviors were anticipatory or abnormal, further comparisons were made between before and after a show.

### 4.2 Show Birds: Before vs. After Shows

Before‐and‐after show comparisons were analyzed to provide further context for all the behavioral categories observed, particularly the abnormal behaviors exhibited by the birds participating in the free‐flight show. Abnormal behaviors were most prevalent before shows, however, significantly reduced after shows, which could be indicative of a potentially anticipatory effect of the bird show itself (Clegg et al. 2018; Krebs et al. 2017). Anticipatory behaviors precede a predictable event (inclusive of AIPs, such as shows and presentations) that is perceived as a positive experience by the animal, such as enrichment, staff or visitor interaction, or feeding opportunities (Spruijt et al. 2001; van der Harst et al. 2003). As previously mentioned, the free‐flight program meets the requirements of an anticipatory event as it is a scheduled activity that occurs daily and provides opportunities such as food and staff interactions. The significant reduction in abnormal behaviors post‐presentation further suggests that these behaviors met the criteria of pre‐presentation anticipatory behavior. If the behavior was purely abnormal, it would be consistent regardless of time (i.e., the prevalence of abnormal behaviors pre‐show would not be significantly different post‐show). As the abnormal behaviors significantly reduced post‐show, this could be indicative that these behaviors may be anticipatory in nature and related to food‐ or staff‐seeking behaviors.

The anticipatory impacts that AIPs could potentially produce have been mostly limited to mammalian species, with minimal literature existing outside of this taxon (Miller et al. 2011; Jensen et al. 2013; Watters 2014; Krebs et al. 2017). Our study demonstrated similar trends in which anticipatory behaviors (which in our study were termed “Abnormal”) increased pre‐show and decreased post‐show. Outside of AIPs, anticipation has been observed in zoo settings prior to fixed‐feeding schedules and the arrival of staff, which further suggests that the increase in abnormal behaviors prior to shows are anticipatory (Brando et al. 2019; Carlstead 2009; Anderson et al. 2020; Fernandez 2021).

Social behaviors were significantly higher pre‐presentation which may be due to choice‐based training sessions and other husbandry procedures occurring during this period with staff. Birds that participate in AIPs can be potentially exposed to increased opportunities to exhibit social behaviors between staff and possibly conspecifics when compared to non‐show (regularly housed) birds. For instance, in a standard aviary, staff often enter once or twice a day for general cleaning and feeding, whereas birds involved in AIPs will have staff regularly visiting and interacting with them throughout the day. An increase in social behaviors could indicate that birds show and presentations have potential enriching welfare impacts (Engebretson 2006; Nicol and Pope 1993; Seibert 2006). In addition, social behaviors are critical to psittacines as they are often a paired or flocking species; parrots housed in inadequate environments with limited opportunities to socialize have been correlated with behavioral issues such as self‐mutilation and feather plucking (M. P. Jones 2001; Park 2003; van Zeeland et al. 2009; Mellor et al. 2018). However, raptors are primarily solitary or pair‐bonded birds when compared to parrot species, and while this has not been investigated broadly in zoo‐housed raptors, it may have differing welfare impacts for these species (Negro and Galván 2018).

In addition, there was a significant increase in grooming and inactive behaviors and a nonsignificant increase in foraging behaviors post‐presentation. The increase in inactivity post‐presentation is consistent with Fernandez et al. (2021) and inconsistent with other zoo AIP welfare literature, which observed an increase in activity as well as species‐typical behaviors post‐presentation (Miller et al. 2011; Jensen et al. 2013). The increase in foraging and grooming behaviors post‐presentation could suggest a positive impact, as increases in species‐typical behaviors are commonly associated with improved welfare—especially if it is reflective of the species' wild counterpart (Hill and Broom 2009; Tallo‐Parra et al. 2023; Wolfensohn et al. 2018). However, this needs to be interpreted cautiously as there were many different species of birds involved in this study that could have different species‐typical behaviors and ecologies. For instance, parrots are largely diurnal, whereas raptor species can differ between diurnal and nocturnal. The diurnal species within this study (sooty owl and barking owl) were involved in the free‐flight program, which occurred during the day, which may affect their welfare states in ways that this study did not measure.

### 4.3 Show Birds vs. Non‐Show Birds

Show birds had a significant reduction in inactivity compared to non‐show (regularly housed) birds, which mimics what would be observed in wild settings for parrots. Wild parrot species do not spend substantial portions of their time exhibiting sedentary behaviors; rather, they engage in other active behaviors, such as foraging (Rose et al. 2017; Rozek et al. 2010;). This suggests that there could be a potentially enriching impact of participating in presentations that reflects the foraging activity of wild parrot species (Rachinas‐Lopes et al. 2024; Rose et al. 2018; Woods et al. 2022). However, while inactivity decreased, active behaviors only approached significance, and no species‐typical behaviors increased, which may indicate overly minimal or neutral welfare impacts. Furthermore, as there were no raptor species that were non‐show birds due to the limitations of the facility, this comparison may only be relevant to parrot species. This difference could also have been due to the increase in “Other” (out‐of‐sight) behaviors, due to show birds being sporadically removed for husbandry and training procedures.

Non‐show birds exhibited higher grooming and foraging behaviors, which are typically indicative of positive welfare as they are species‐typical across all bird species (Rozek et al. 2010; Rachinas‐Lopes et al. 2024). However, the housing conditions may have influenced the results, as conspecifics were housed together regardless of whether they were involved in the show or not. Non‐show birds were provided with enrichment devices and fresh browse whilst the show birds were partaking in the public presentation, which may have provided more opportunities to engage with the devices. In addition, show birds received more opportunities to forage outside of the observation period, such as during the free‐flight show, as they would receive small amounts of their daily food intake during presentations. Both factors may contribute to explaining the significant increase in foraging and grooming behaviors observed in the non‐show birds, as well as the increase in “Other” behaviors in show birds. These findings are not consistent with other AIP zoo welfare literature, which has observed an increase in species‐typical behaviors in presentation animals, typically post‐show (Trone et al. 2005; H. Jones et al. 2016; Orban et al. 2016; Delfour et al. 2020). However, it is difficult to compare results as most of these papers did not compare show to non‐show animals but rather a pre‐ and post‐behavior comparison. In addition, studies do not consider species differences, and none of the aforementioned investigated birds.

To provide further context to the potential welfare implications of public presentations, both behavioral (ethogram) and physical (body and FCS) measures were compared between show birds and non‐show birds. Show birds exhibited significantly less abnormal and inactive behavior compared to non‐show birds, which could highlight a potential enriching effect of presentations. BCS was closer to ideal in show birds compared to non‐show birds. Non‐show birds were slightly under ideal (skinnier), which could indicate that they were slightly underweight or had a lower general muscle mass. Flying is a critical species‐typical behavior for birds that is necessary to maintain muscle mass and general health (Biewener 2011; Peng et al. 2013). Restricted flight has also been associated with an increase in abnormal behaviors amongst birds and decreased bone density (Engebretson 2006; Aguado et al. 2015). In addition, a higher prevalence of osteoporosis has been observed in domestic fowl with restricted flight access (Peng and Broom 2021; Ebisawa et al. 2021; Xu et al. 2022). Participating in a public flight presentation could potentially provide more opportunities to exercise and exhibit species‐typical flight behaviors without the restraint of an aviary, which subsequently could improve welfare (Woods et al. 2022; Rachinas‐Lopes et al. 2024).

### 4.4 Limitations and Future Research

BCS and FCS are typically assessed utilizing visual and tactile methods. However, to reduce the stress of handling the birds, these were only scored visually, which may have altered results. BCS and FCS are not well‐documented in zoo welfare research and have yet to be adopted widely within peer‐reviewed zoo welfare literature. However, while some zoos do incorporate these assessments into their practice, these are not often utilized within research. Furthermore, these measures can be subjective, hence why weight is typically used instead or in combination to provide further context. In addition, the musculoskeletal structure and anatomy of raptor species, especially regarding pectorals and wing architecture, differ when compared to parrot species, which would alter the general BCS of the show‐birds due to anatomical differences (Corvidae et al. 2006). Although this was attempted to be mitigated through the use of a second observer and assessing inter‐observer reliability, future research should design a BCS based on Orders as opposed to a generalized chart as this study did. A BCS for each Order may be more applicable for accounting for species differences; however, it can make interpretation of results and broader applicability difficult.

This study was also limited by the number of individuals: a total of 10 birds across 7 species were observed, of which there were only non‐show parrots and no non‐show raptor species, which limited the analysis and results of this study. Although multiple data points per individual were recorded, this does not resolve the issue of a limited sample size. Furthermore, the groupings of the birds were determined by the facility based on current housing situations. Conspecifics were housed together for welfare reasons and, as such, non‐show birds received more time per individual engaging with staff as well as feeding and enrichment opportunities compared to birds housed in regular zoo aviaries. Future studies should observe birds that are not housed with presentation conspecifics, as this could present differing results—particularly regarding grooming and foraging behaviors, which may be lower in non‐show birds than observed within this study due to the increased opportunity to interact with staff, food, and enrichment that the non‐show birds had while being housed with their show conspecifics.

Although this paper did utilize more than one welfare measure (behavioral and physical), further research should continue to integrate the use of multiple measures when assessing animal welfare to ensure a more comprehensive analysis (Botreau et al. 2007; N. Jones et al. 2022). Other behavioral, physical, and physiological measures that could have been implemented include behavioral diversity, weight, and corticosterone levels. Using a combination of behavioral, physical, and physiological measures would ensure a well‐rounded and comprehensive welfare assessment compared to single measures (Brereton 2020; Fernandez and Harvey 2021).

It is also crucial to note that while the outcomes of this study had an overly neutral and potentially positive welfare impact on the birds involved in presentation AIPs, this should not be used as a means to justify all AIPs. A lack of negative welfare indicators should not determine whether AIPs are beneficial to the animal; rather, positive indicators of welfare, including an increase in species‐specific behaviors or a reduction in abnormal or stress‐related behaviors, should be sought out instead (Spooner, Jensen, et al. 2021). Rather than focusing on eliminating negative experiences, zoo AIP welfare research needs to move to enhancing or forming positive experiences and assessing welfare based on those positive indicators. As such, zoo AIP welfare literature should move toward investigating how to make an AIP the most beneficial and least impactful for the animals involved, rather than simply assessing or minimizing potential welfare impacts.

### 4.5 Significance

This study provides empirical evidence in a severely understudied area regarding the welfare impacts of animals involved in AIPs within zoological facilities. Birds have been one of the least investigated taxa within zoo welfare literature, and while this has been emphasized in past literature and is currently in the process of being rectified with current and future research, there is still a severe disparity within the current literature. Birds are one of the most common species involved in AIPs worldwide. Similarly, AIPs occur in over 75% of zoos, yet less than 2% of published zoo welfare studies measured the effects of AIPs such as ambassador, presentations, or show programs (Binding et al. 2020; D'Cruze et al. 2019). This research contributes to providing a better understanding of the impacts that presentations could have on bird welfare, enabling zoos to refine and tailor these experiences to better meet the species‐specific welfare needs of the birds in their care.

This research provides a basic framework that can be utilized to investigate and assess welfare across various types of AIPs, species, and facilities. This could assist in selecting individuals for AIPs based on similar or more elaborate welfare assessments. This research also aimed to improve our knowledge and understanding of AIPs and how they impact animal welfare. It is well documented how AIPs improve visitor learning; however, there is not the same depth of research in regards to the animal welfare impacts of being involved in an AIP. If visitors also understood the potential welfare impacts, they may be more inclined to support, participate, and advocate for these programs.

## 5 Conclusion

Although current literature has explored the visitor experience regarding AIPs, AIPs and their impact on animal welfare have not been investigated to the same extent. This research suggests that presentations have overall minimal welfare impacts and no negative welfare impacts. There may be a potentially enriching effect on zoo‐housed birds involved in AIPs; however, this may be species‐specific and may not be applicable to each of the species involved in the study. In addition, this study highlights the importance of providing context and multiple measures when assessing welfare. Although this research did not exhaust the number of measures that could have been used, limited context‐specific data may not account for other factors that may be influencing behavior. This was evident between event and non‐event comparisons, which did not account for anticipatory effects.

## Data Availability

The data that support the findings of this study are available from the corresponding author upon reasonable request.

## Appendix 1

The form used to log participation in the free‐flight show for each of the show birds. The bird's name and species were written on the *y* axis and the correlating days and on the *x* axis. Staff members would tick or cross in each box dependent on participation in the show.
